# Reduction of arsenic content in a complex galena concentrate by *Acidithiobacillus ferrooxidans*

**DOI:** 10.1186/1472-6750-4-22

**Published:** 2004-10-13

**Authors:** Mario Makita, Margarita Esperón, Benito Pereyra, Alejandro López, Erasmo Orrantia

**Affiliations:** 1 Centro de Investigación en Materiales Avanzados, Miguel de Cervantes 120, 31109 Chihuahua, México; 2 Instituto Tecnológico de Chihuahua II, Chihuahua, México; 3 Universidad Autónoma de Nuevo León, Monterrey, México

## Abstract

**Background:**

Bioleaching is a process that has been used in the past in mineral pretreatment of refractory sulfides, mainly in the gold, copper and uranium benefit. This technology has been proved to be cheaper, more efficient and environmentally friendly than roasting and high pressure moisture heating processes. So far the most studied microorganism in bioleaching is *Acidithiobacillus ferrooxidans*. There are a few studies about the benefit of metals of low value through bioleaching. From all of these, there are almost no studies dealing with complex minerals containing arsenopyrite (FeAsS). Reduction and/or elimination of arsenic in these ores increase their value and allows the exploitation of a vast variety of minerals that today are being underexploited.

**Results:**

Arsenopyrite was totally oxidized. The sum of arsenic remaining in solution and removed by sampling represents from 22 to 33% in weight (yield) of the original content in the mineral. The rest of the biooxidized arsenic form amorphous compounds that precipitate. Galena (PbS) was totally oxidized too, anglesite (PbSO_4_) formed is virtually insoluble and remains in the solids. The influence of seven factors in a batch process was studied. The maximum rate of arsenic dissolution in the concentrate was found using the following levels of factors: small surface area of particle exposure, low pulp density, injecting air and adding 9 K medium to the system. It was also found that ferric chloride and carbon dioxide decreased the arsenic dissolution rate. Bioleaching kinetic data of arsenic solubilization were used to estimate the dilution rate for a continuous culture. Calculated dilution rates were relatively small (0.088–0.103 day^-1^).

**Conclusion:**

Proper conditions of solubilization of arsenic during bioleaching are key features to improve the percentage (22 to 33% in weight) of arsenic removal. Further studies are needed to determine other factors that influence specifically the solubilization of arsenic in the bioleaching system such as: pH, dissolved oxygen concentration, redox potentials, nature of concentrate and temperature among others. *At. ferrooxidans *was able to completely oxidize the minerals present during the arsenic bioleaching. Other elements present originally in the concentrate such as Zn, Sb, and Cu were also solubilized. The process of bioleaching is expected to be influenced by mechanisms that still need to be established due to the diversity of the minerals involved and by the presence of traces of metals in the concentrate. The increase in pulp density generates a decrease in the dissolved arsenic concentration. This decrease is greater in runs where air was not injected to the system. The maximum rate of arsenic dissolution in the concentrate was found using; small surface area of particle exposure, low pulp density, injecting air and adding 9 K medium to the system. The effect of addition of ferric chloride during the arsenic bioleaching resulted in a decrease of the solubilized arsenic in the system. The presence of CO_2 _is associated to the decrease in arsenic dissolution.

## Background

The term bioleaching refers to the bacterial conversion of an insoluble metal (usually a metal sulfide, e.g., CuS, NiS, ZnS) into a soluble form (usually the metal sulfate e.g., CuSO_4_, NiSO_4_, ZnSO_4_). When this happens, the metal is extracted into water [1,2]. The first bacterium discovered that was able to oxidize minerals was *Acidithiobacillus ferrooxidans *(*At. ferrooxidans*, previously *Thiobacillus ferrooxidans*), a gram-negative, acidophilic, chemolithoautotrophic, non-spore forming rod. Nutritionally, typical *At. ferrooxidans *isolates are considered obligate autotrophs. *At. ferrooxidans *is able to use either ferrous iron or a wide variety of reduced inorganic sulfur species as an electron donor compounds. It is also able to grow using ferric iron as an electron acceptor, provided that an electron donor, such as reduced inorganic sulfur compound is present. Energy is derived from the oxidation of reduced iron and sulfur compounds, including ferrous ion, sulfide, elemental sulfur and thiosulfate, with final oxidation products being ferric ion and sulfate [3,4]. Other microorganisms considered important in commercial mineral biooxidation processes are: *Acidithiobacillus thiooxidans*, *Acidithiobacillus caldus*, *Leptospirillum ferrooxidans*, *and Acidiphilium acidophilum *[3].

Bioleaching has emerged as a simpler, safer, and less expensive process than other alternatives for most limestone, granitic, or other host rocks that have secondary replacement of pyritic minerals containing metal values. In recent years, biooxidation has shown itself to require less capital, reduced operating cost, and less skilled operating and maintenance personnel than the traditional pressure oxidation or roasting techniques [5].

This technology has been used for treating specific mineral ores, mainly copper and gold bearing ores [6-8]. Moreover, bacterial leaching in acid medium has been successfully applied in: uranium metallurgy [9]; silver, gold and lead recovery [10]; zinc [11]; and new processes have been developed for cobalt recovery [12,13].

A complex sulfide ore is an association of galena (PbS), sphaelerite (ZnS) and chalcopyrite (CuFeS_2_), disseminated in a pyritic matrix. Besides of lead, zinc and copper as valuable metals, such deposits may contain significant quantities of silver, gold, arsenic, antimony, bismuth and mercury. Numerous economically important deposits of these ores exist in the world [14].

Complex ores are often characterized by particularly fine intergrowth of the mineral values. Due to these specific mineralogical characteristics, it is necessary to finely grind and concentrate the ore prior to the solubilization of the valuable metals. To obtain separate concentrates by selective flotation involves high unit-cost, poor quality of the concentrates and relatively low overall recoveries [15].

Arsenic is a major impurity present in numerous sulfide deposits. The presence of arsenic in mineral concentrates drastically diminishes their value, and results in two types of problems. On one hand, arsenic produces metallurgical problems, making difficult the metal extraction and the recovery of a final product of high purity. On the other hand, arsenic is regarded as a highly toxic contaminant resulting in environmental problems due to its atmospheric release and possible water contamination associated to the processing of arsenic bearing ores and concentrates [16].

Furthermore, this element is generally toxic for microorganisms and its dissolution could inhibit bacterial activity in the bioleaching process. It has been shown that high concentrations of arsenic in solution inhibit bacterial growth, with As(III) reported to inhibit bacteria to a greater degree than As(V) [17].

The processing of complex arsenic bearing ores and concentrates requires a good understanding of the mechanisms involved previous to the design and development of an adapted sequence of process units. In particular, a complete chemical and mineralogical characterization of the ore is essential from both biological and metallurgical points of view in order to determine the possible inhibition problems and the requirement for effluent treatment processes [12].

The interest in the study of bioleaching of arsenic-containing minerals, started two decades ago with a publication dealing with the degradation of arsenopyrite (FeAsS) through *At. ferrooxidans *in gold-arsenic concentrates. This study established the economic feasibility of this technology and its potential for alleviating some environment related problems [18]. Later, other researchers [19] studied the physical changes occurring in the mineral-bacteria interphase in pure crystals of arsenopyrite. They found that bacterial oxidation is characterized by three stages that coincide with the phases of the general bacterial growth and that the amorphous arsenates produced are deposited over the crystal surface, thus interrupting the bioleaching process.

There have been other studies aimed to determine the influence of several factors over the arsenic bioleaching in gold minerals and concentrates. These studies have been mainly focused to maximize gold extraction and considered bioleaching as a pretreatment in the cyanidation process, consequently leaving the importance of arsenic dissolution and extraction in a second term.

Ubaldini *et al *[20] studied the arsenopyrite bioleaching over a refractory mineral using a mixture of *At. ferrooxidans *and *At. thiooxidans*. They achieved an increase in gold extraction from 55.3% to 96.8% using the following conditions; pulp density 20%, pH 2, stirring conditions 200 rpm, temperature 30°C, time 7 days. Another research group used a domestic strain of *At. ferrooxidans *and a gold refractory mineral to study the influence of a magnetic field, surfactant addition and the presence of Ag^+^, Bi^3+^, Co^2+ ^y Hg^2+ ^ions over the bioleaching process [21]. They found a reduction to 120 hours to leachate 60% of arsenic using magnetized water and the addition of tween-80 surfactant and Ag^+ ^ion [21].

As indicated above, previous studies are focused in using bioleaching as a technique to eliminate arsenic in high-value minerals such as gold concentrates. However, there is no reported work on arsenic elimination or reduction in minerals of low value such as lead.

This work is aimed to reduce the arsenic content in complex concentrates of galena and to generate preliminary data to allow us to conduct further studies to understand the complex behavior of the bioleaching process. This preliminary study deals with the influence of main factors in batch bioleaching over the arsenic solubilization from a complex lead concentrate.

The main factors influencing the biooxidative treatment were tested using a two level fractional factorial plan of experiments (2^7–4^), and they were: surface area, pulp density, carbon dioxide bubbling, air bubbling, 9 K medium addition, FeCl_3 _addition, and two different *At. ferrooxidans *strains.

## Results and discussion

### Chemical and mineralogical characteristics of the concentrate

The chemical composition of the concentrate, determined by atomic absorption spectrometry (AAS) and AAS Hydride System for arsenic was: 3.85% As, 14.75% Fe, 23.53% S, 49.76% Pb, 2.48% Zn, 0.51% Sb, 0.29% Cu, 0.28% Bi. The remaining 4.56% being other elements such as: Cd, Ca, Ag, K, Mn, Na, Ni, Ba, Mo, Sn, Si, O.

Table 1 shows major phases in the concentrate determined by X-ray diffraction (XRD).

**Figure 1:**
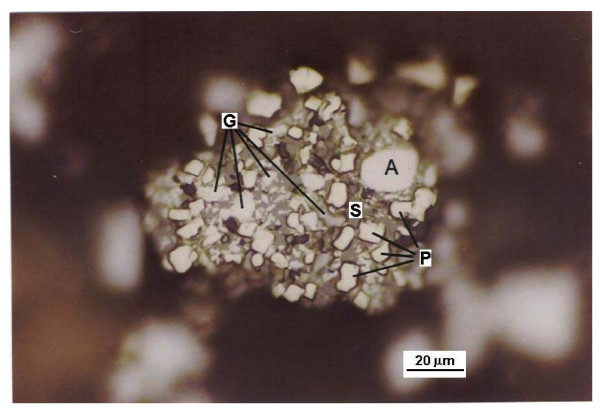
**Photography of a site in surface of a mount **G: Galena; S: Sphaelerite; A: Arsenopyrite; P: Pyrite

**Figure 2:**
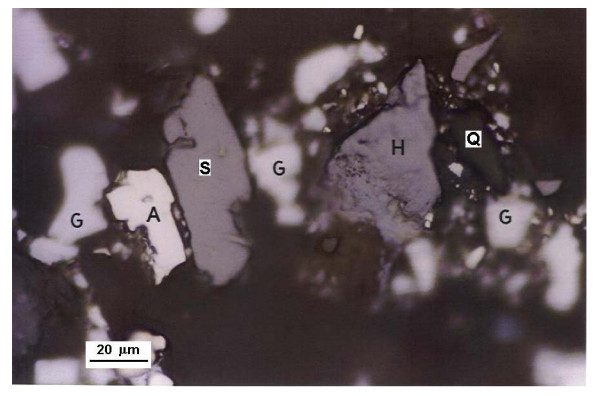
**Photography of a site in surface of a mount **G: Galena; S: Sphalerite; A: Arsenopyrite; H: Hematite; Q: Quartz

**Figure 3:**
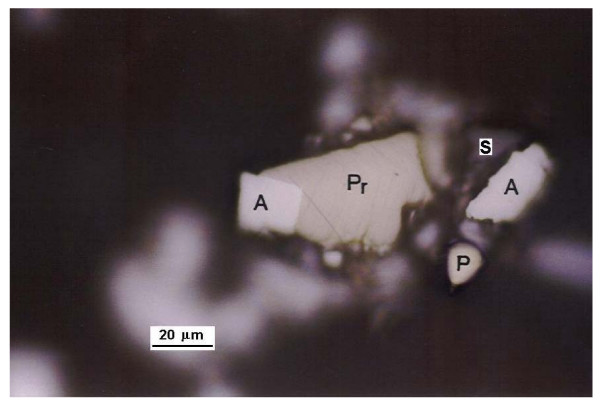
**Photography of a site in surface of a mount **S: Sphaelerite; A: Arsenopyrite; P: Pyrite; Pr: Pyrrotite

Photographs of prepared mounts (Figures 1, 2 and 3) show several mineral species and some mineral associations found in the concentrate.

Studies dealing with bioleaching through *At. ferrooxidans *have provided great amount of basic knowledge about this process and have been useful in the understanding of the physicochemical and microbiological aspects of this phenomenon [3,22].

Biooxidative dissolution of arsenopyrite [19,23-29] and the parameters for adapting the bacteria to arsenic have also been studied [17,30]. However, a major disadvantage of these studies is the fact that they were performed using chemicals reagents or pure crystals of pyrite and/or arsenopyrite, synthetic or manually sorted with the aid of a microscope and therefore, they do not represent the conditions and complexity involved in the bioleaching treatment of concentrates.

There are only a few of studies dealing with arsenic-bearing ores and concentrates of natural sources [12,14,31,32]. It is desirable that the trend of today's studies of bioleaching of natural ores be aimed to understand the factors influencing these phenomena if this technology is expected to reach industrial importance. However, it is clear that the complexity of some sulfide minerals are due to the association among species and to the coexistence of inclusion forms of many of these sulfide materials. These two issues (complexity and inclusions) are important factors that may inhibit the process and produce non-successful intrinsic behavior of the bioleaching mineral systems [33].

The concentrated used in this study was highly complex as can be seen in Figures 1, 2 and 3. Also in these Figures the relative composition of the mineral species and their associations described in Table 1 are shown. The complex array of compositions showed in these Figures means that the process of bioleaching is expected to be influenced by mechanisms that still need to be established by the diversity of the mineral species involved and by the presence of traces of metals in the concentrate.

### Mineral species oxidized

**Figure 4:**
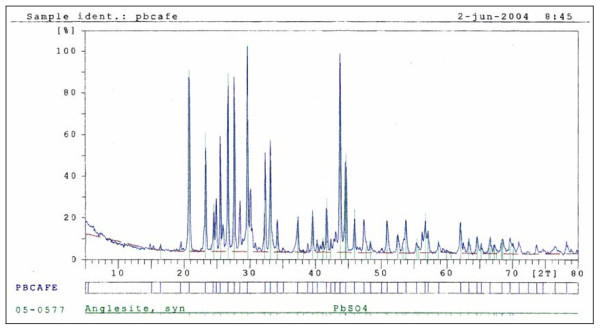
XRD of the residual solid from bioleaching

**Figure 5:**
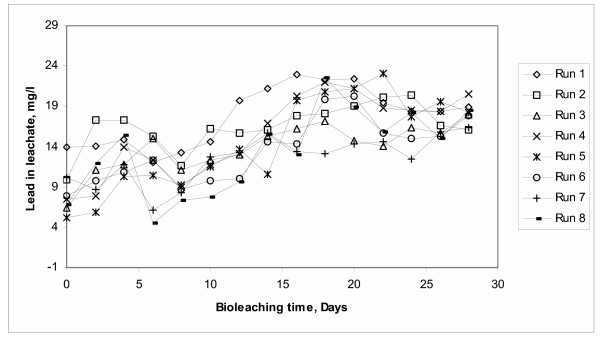
Lead Solubilized

XRD analysis of the residual solid from bioleaching (Figure 4) shows only the presence of crystalline phase anglesite (PbSO_4_). Chemical analysis performed through AAS resulted in the presence of residual arsenic. This indicates that all crystalline species present in the concentrate were completely oxidized through the bioleaching process. This means that all arsenic present in arsenopyrite was either solubilized (22 to 33%) or precipitated in amorphous compounds (67 to 78%). Also, galena (PbS) was completely oxidized to anglesite. Only a very small portion of anglesite remained in solution (25 mg/L, see Figure 5) while, the main phase of this appeared as a solid precipitate. Other elements present originally in the concentrate such as Zn, Sb, and Cu were also solubilized. This fact opens a window of a great potential for separating lead from other elements that often are present along in the mineral concentrates [2].

### Formation of precipitates during biooxidation

Formation of precipitates of arsenic and iron has been commonly observed with the use of *At. ferrooxidans *[34]. Several other compounds have also been observed in solid state during the bioleaching of arsenopyrite, among these are; ferric arsenate, elemental sulfur, amorphous ferric arsenate FeAsO_4_^·^*x*H_2_O, jarosite KFe_3_(SO_4_)_2_(OH)_6 _and scorodite FeAsO_4_^·^2H_2_O [19,25-27].

It has been reported that the elemental sulfur and ferric arsenate are accumulated in the surface of the grains forming a coating barrier that avoids the contact between the mineral and the Fe^+3 ^ions in the indirect oxidation, inhibiting the leaching of the mineral species [19,27]. The formation of precipitates of the jarosite-type are prevented by a pH control to levels lower than 1 [33], while the formation of S^0 ^can be avoided by the use of bacterial consortia in which sulfur-oxidant bacteria such as *At. caldus *[28] or *At. thiooxidans *are included [3].

In this study the precipitation of amorphous arsenic compounds was important during the bioleaching process. In samples taken at the end of the experiment there was a difference in the arsenic content in the digested mineral residue, which was treated by a hydrochloric acid digestion, to the non-digested residue (see Table 2). This last, suggests the presence of two types of arsenic compounds in the residue, probably amorphous ferric arsenates and jarosite-type precipitates due to the acidic conditions used in the experiment (pH ≥ 2). However, other studies are needed using this kind of concentrates in order to determine the type and amount of precipitates formed and the kinetics involved in this process.

### Significant factors in arsenic dissolution

**Figure 6:**
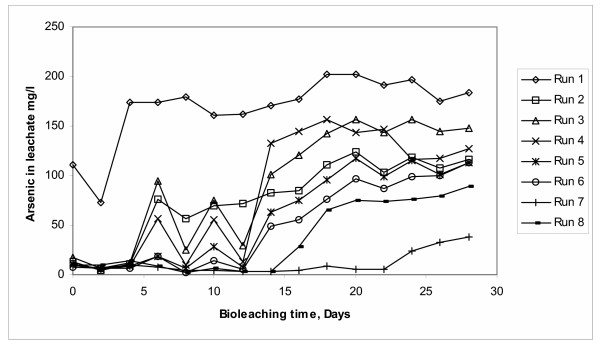
Arsenic Solubilized

Arsenic bioleaching results for the eight experimental runs (Table 3) are shown in Figure 6. In this plot a great deal of scattered data among experimental runs is observed, this means that still a great deal of variability exists among the effect of the factors considered in this study.

Table 4 shows the results of the least-squares-multiple-regression model fitting to arsenic dissolution data using time and factors as explanatory variables. With the exception of *At. ferrooxidans *strains, all of the other factors resulted to be highly significant in its influence to the arsenic dissolution. The resulting model was the following (Minitab 13.0):

Arsenic = 52.0 - 66.3 Pulp Density - 32.6 Surface Area - 21.1 Ferric chloride - 12.5 Carbon dioxide + 29.3 Air + 29.3 9 K Medium + 3.00 Strain + 3.94 Days.     (1)

With a determination coefficient of R^2 ^= 0.834

The negative sign in some of the variables of the model indicates that in order to maximize bioleaching of arsenic, these factors must be kept in low levels. These were denoted with a zero value in the model. Thus, the levels of factors that should be considered in the model are the following: pulp density at 10%, low surface area, no FeCl_3 _and CO_2 _addition, air and 9 K Medium addition and any strain present.

### Best run

One of the main disadvantages for the commercial use of bioleaching is the slow nature of this process, which is due mainly to the relative slow growth rate of the bacteria [5,35], to the long period of time needed for the bacteria to adapt to the mineral environment and particularly to mineral complexes [31] and to inhibition problems due to the products of the bioleaching [36].

In Figure 6, Run 1 presents the highest arsenic concentration in the leachate (approximately 200 mg/L). The combination of the previously established levels of factors for this run are shown in Table 3 and coincides with the required combination of levels to maximize the arsenic dissolution as stated in the model of the above Equation (1).

**Figure 7:**
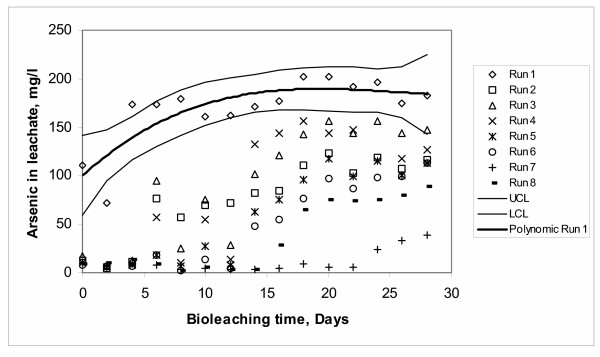
Third order model fit and confidence interval for Run 1

The graphical pattern of the arsenic bioleaching for Run 1 (Figure 7) shows some features that are potentially adequate for the use of this combination in reduction of arsenic content in future studies. Some of these features are: the lack of an adaptation period (lag-time), a very pronounced slope and that the stability of the system is reached in a relatively short time (four days) compared to the other experimental runs.

The data for this run (Run 1) were fitted through a third order polynomial linear regression model and statistically analyzed over a 95% confidence interval (see Figure 7). The model of arsenic present in solution in Run 1 is the following (Minitab 13.0):

Arsenic = 100.16 + 11.487 Days - 0.4705 Days^2 ^+ 0.006 Days^3 ^    (2)

With a determination coefficient of R^2 ^= 0.6585

Besides of all these convenient features in Run 1, the percentage of bioleached arsenic is still low. Proper conditions of solubilization of arsenic during bioleaching are key feature to improve the percentage (yield) of arsenic removal. Therefore, further studies are needed in order to determine the other factors (not considered in this work) that influence specifically the solubilization of arsenic in the bioleaching system such as: pH, dissolved oxygen concentration, redox potentials, nature of concentrate and temperature among others.

### General evolution of arsenic in the leachate

**Figure 8:**
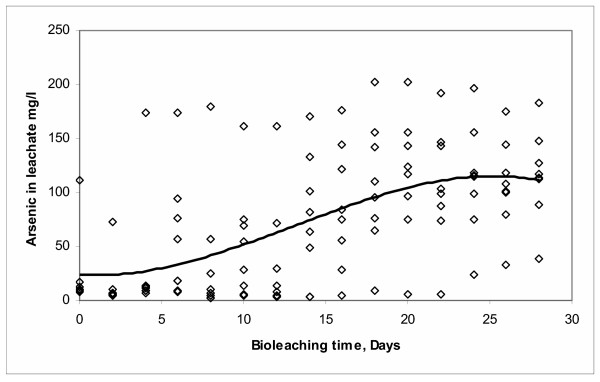
Third order model fit for all data

When time (days) was used as the only independent variable, data collected in all runs were adjusted to a third order polynomial regression model and the analysis is shown in Figure 8. The resulted general equation of this model is the following (Minitab 13.0):

Arsenic = 23.888 - 1.0597 Days + 0.5161 Days^2 ^- 0.0131 Days^3 ^    (3)

The determination coefficient of this model was R^2 ^= 0.3237, which represents a value relatively low due to the absence of the other significant factors (pulp density, CO_2_, 9 K medium, particle size, air injection and ferric chloride added).

It is worth to note that this model resembles the well known general three-phase bacterial growth model.

### Effect of pulp density

**Figure 9:**
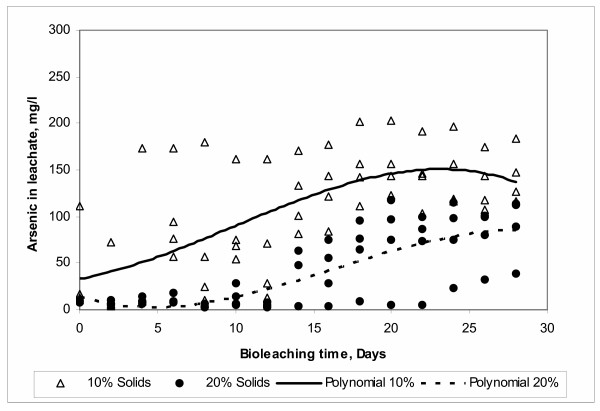
Effect of Pulp Density

The pulp density effect is shown in Figure 9. Here a greater arsenic dissolution is achieved with a low level of 10% of solids. This result is in agreement with results reported by other researchers working with complex concentrates of copper and zinc [14], complex sulfides of copper, lead and zinc [37] and copper concentrates [38,39]. In all of these studies greater levels of leaching were achieved with low pulp densities. The reduction in the bioleaching rate can be due to the fact that at higher concentrations of solids causes an increase in the friction between particles, and probably avoiding the adhesion between the particle and bacteria [40]. This friction may consequently cause some mechanical damage to the cell [41].

In the case of arsenic, it is possible that a high pulp density may cause a greater interaction between the dissolved arsenic and other products of biooxidation. Thus, increasing the rate of formation of precipitates and consequently decreasing the concentration of arsenic in solution.

**Figure 10:**
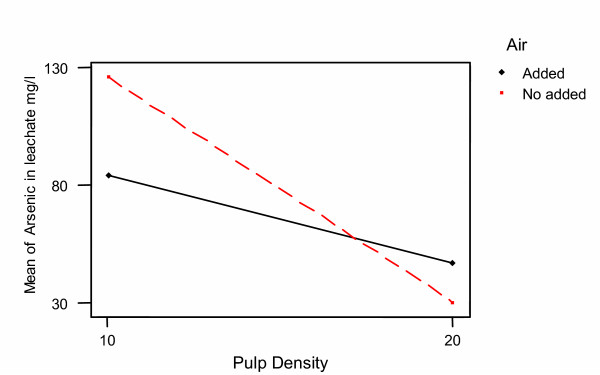
Interaction plot Pulp density – Air

In our study it was found a strong interaction between the pulp density and the air injection with respect to the arsenic removal. The ANOVA analysis of this interaction is shown in Table 5, and the plot in Figure 10. The increase in pulp density generates a decrease in the dissolved arsenic concentration in all runs. However, this decrease is greater in runs where air was not injected to the system.

### Effect of surface area

**Figure 11:**
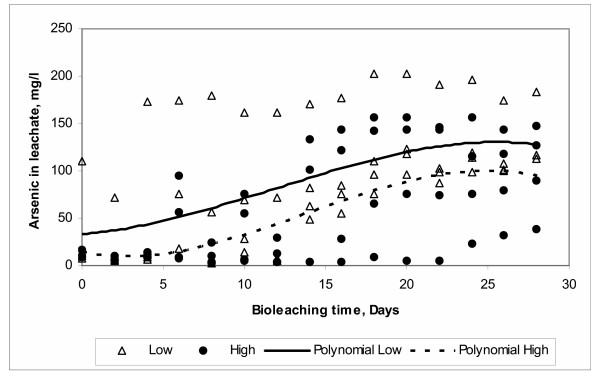
Effect of Surface Area

The effect of surface area (particle size) can be observed in Figure 11. Here a greater arsenic dissolution is presented when the particle surface area is small, in other words, where bigger particles are present in the system. One would expect that a greater exposed surface area to the microbial attack would reflect an increase of the arsenic dissolution. However, this unexpected result is in agreement with other studies reported in the literature [42,43]. Explanations for this phenomenon are in the sense that it is possible for the bacteria to preferentially attack some sites formed during the solids grinding process [42]. Other study of the leaching of chalcopyrite reported that the rate of leaching increased with the use of larger particles sizes [42]. They suggested that this was due to a greater efficiency of bacteria attachment to the particles. Other explanations are focused on disregarding the associated physical factors such as surface area and stressing the specific and non specific interactions. Among the specific are the ionic and hydrogen bonds, and chemical and protein interactions. Non-specific are hydrophobic interactions such as surface free energy and electrostatic [44].

A possible explanation for the behavior observed in this study is based in the great complexity of the mineral concentrate that may enhance the specific interactions. These interactions are affected by the particles of smaller sizes where the greater surface area leaves each particle exposed to a greater amount of different mineral species. These species increase the complexity of the medium thus affecting directly the solubilization of arsenic during the bioleaching. The complexity of the medium due to small size particles can be further increased when a high pulp density is used as pointed out in the previous section. These two issues, complexity and pulp density, combined in the conditions above mentioned may produce a negative effect over the arsenic dissolution.

### Effect of ferric chloride

**Figure 12:**
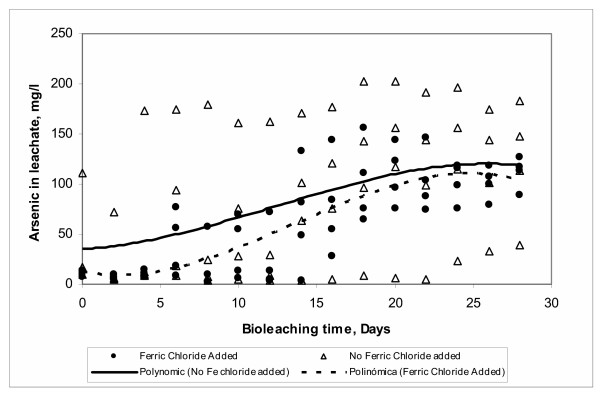
Effect of Ferric Chloride

In the present study the effect of addition of ferric chloride during the arsenic bioleaching resulted in a decrease of the solubilized arsenic in the system as observed in Figure 12. Information on this effect is limited in the literature with only some studies reporting the influence of the ion chloride on the sulfur and iron oxidation during bioleaching of some chemical species using *At. ferrooxidans *and /or *At. thiooxidans *[45-47].

### Effect of carbon dioxide

**Figure 13:**
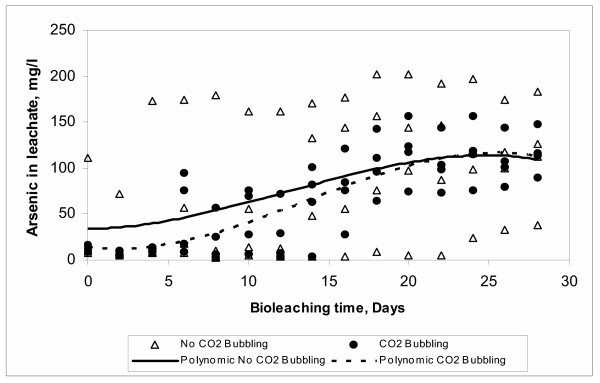
Effect of Carbon Dioxide

It is well known that autotrophic organisms such as *At. ferrooxidans *require CO_2 _for its growth. This is due to the fact that these bacteria are able to use CO_2 _as its only source of carbon for their biosynthetic reactions. CO_2 _fixation is usually achieved through the so-called Calvin-Benson-Bassham cycle [48]. In Figure 13 it can be seen that the arsenic dissolution is reduced when CO_2 _is bubbled into the system. This behavior is in agreement with studies reported in literature that established that the consumption of carbon by the bacteria decreases at high CO_2 _concentrations implying the formation of intracellular bicarbonates [49]. Other authors coincide in that the amount of CO_2 _present in the air is enough to withstand the bacterial growth [14,25].

**Figure 14:**
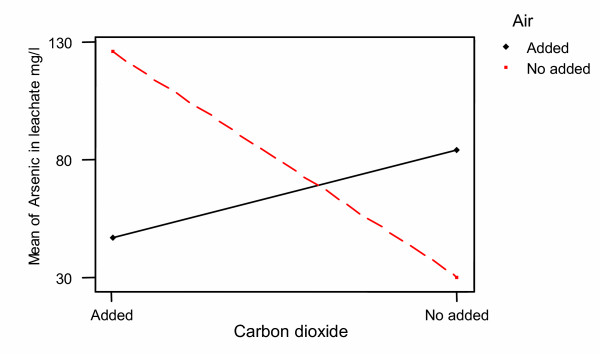
Interaction plot Carbon dioxide – Air

In our study, it was found a strong interaction between the two levels of CO_2 _and air into the system. The ANOVA analysis of this interaction is also shown in Table 6 and Figure 14. However, in our study the presence of CO_2 _according to the results shown in Figure 13 is associated to the decrease in arsenic dissolution. At this time, it is difficult to establish the influence of the presence and concentration of CO_2 _on the dissolution of arsenic. Therefore, further studies oriented to establish the relationship between the dissolved oxygen and the concentration of CO_2 _in the system are needed in order to elucidate this effect.

### Effect of air

**Figure 15:**
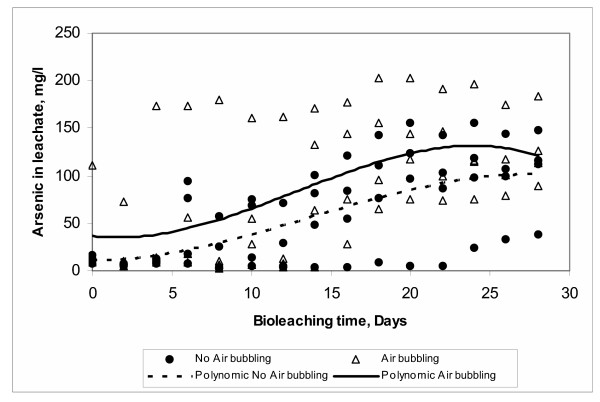
Effect of Air

*Acidithiobacillus ferrooxidans *is an aerobic microorganism that uses oxygen as a final electron acceptor during the oxidation process. However, in absence of oxygen it is still able to grow in the presence of inorganic reduced sulfur compounds by using the ferric ion as an alternative electron acceptor [50]. In these conditions, however, its growth is slower [48]. In the present study, it is not surprising that the arsenic dissolution was increased with the aeration of the system as can be observed in Figure 15. As it was shown previously, the availability of oxygen in the medium decreases when the pulp density increases and also when CO_2 _is admitted into the system. Again a more definite study targeted to measure dissolved oxygen in the system may bring light into the effect of air during the arsenic bioleaching.

### Effect of 9 K medium

**Figure 16:**
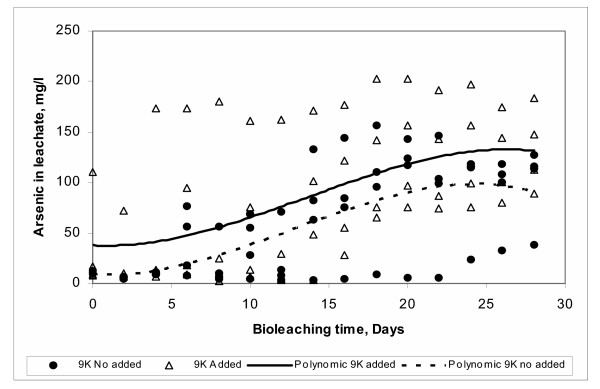
Effect of 9 K medium

The 9 K medium is the source of nitrogen and phosphorous [51] needed for bacterial growth. These two elements are not available in the sulfide minerals. Therefore, they need to be provided during the bioleaching process. Figure 16 shows the effect of the addition of 9 K medium. As expected, the arsenic bioleaching is increased when 9 K medium is used.

### Effect of strain

**Figure 17:**
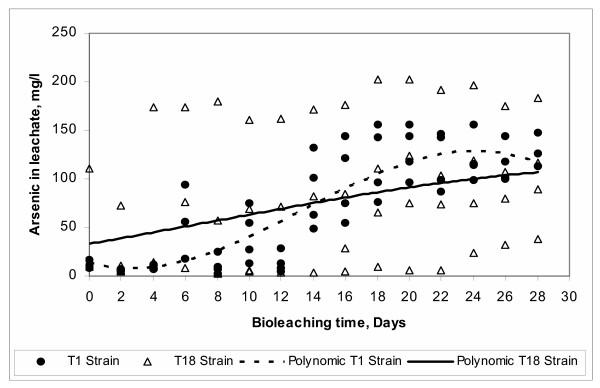
Effect of Strain

In Figure 17 differences of two *At. ferrooxidans *strains used in this study are shown. Strain T1 presents the common bacterial model of growth in three stages, while strain T18 presents a straight model that requires less adaptation time. This can be due to the fact that T18 was previously grown in systematically increased arsenic concentrations as discussed in the material and methods section of this paper.

### Continuous culture dilution rate calculus

**Figure 18:**
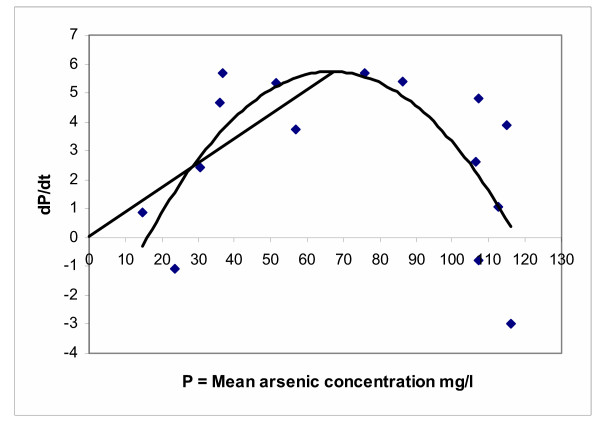
Dilution rate calculus

Data generated from the batch arsenic bioleaching were used as a first approximation for the calculation of the dilution rate D for a future design of a continuous bioleaching system. The procedure used consisted in to derive the third order polynomial equations (2) and (3) with respect to time and plot each equation versus the arsenic concentration in the system. Then to draw a straight line from the origin to the maximum point in each curve (Figure 18). The slope of this line was then defined as dilution rate in agreement with the equation of product balance in a continuous culture vessel in steady state [52,53] as shown in equation (4):


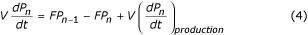


where

*F *= Rate of medium flow through the vessel (volume/time)

*V *= Volume of the vessel (volume)

*D *= F/V Dilution rate (time^-1^)

*P*_*n-1 *_= Inner product concentration

*P*_*n *_= Outer product concentration

*dP*_*n*_/*dt *= Total variation of the product concentration in the vessel

(*dP*_*n*_/*dt*)_*production*_= Product concentration variation due to the production in the vessel

Rearranging Eq. (4):


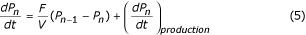


In steady state, *dP*_*n*_/*dt *= 0 and assuming that *P*_*n-1 *_= 0 in a single vessel,


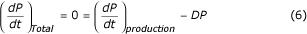


From which


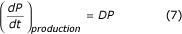


Equation (7) represents a straight line through the origin having a slope of D (dilution rate) and corresponds to the straight line plotted in Figure 18.

Using this method, the calculated dilution rate for the general model of equation (3) is 0.088 days^-1^, while for run 1 in equation (2) is of 0.103 days^-1 ^(not shown in the plot). However, these dilution rates are still relatively small compared to the dilution rates used in industrial scale processes (0.05–0.6 h^-1^) [53].

## Conclusions

Since the bioleaching rate and the levels of leached arsenic are limited (22–33% of the originally present in the concentrate), proper conditions of solubilization of arsenic during bioleaching is the key feature to improve the percentage (yield) of arsenic removal. Therefore, further studies are needed in order to determine the other factors (not considered in this work) that influence specifically the solubilization of arsenic in the bioleached system such as: pH, dissolved oxygen concentration, redox potentials, nature of concentrate and temperature among others.

The performance of the bacteria used in this study (*At. ferrooxidans*) was able to completely oxidize the minerals present during the arsenic bioleaching. Since, galena (PbS) was completely oxidized to anglesite (PbSO_4_) with only a very small portion of anglesite remaining in solution, while the main phase of this appeared as a solid precipitate. Other elements present originally in the concentrate such as Zn, Sb, and Cu were also solubilized.

The complex array of compositions contained in the concentrate, employed in this study, means that the process of bioleaching is expected to be influenced by mechanisms that still need to be established due to the diversity of the mineral species involved and by the presence of traces of metals in such concentrate.

The precipitation of amorphous arsenic compounds was important during the bioleaching process. Results suggest the presence of two types of arsenic compounds contained in the residue, probably amorphous ferric arsenates and jarosite-type precipitates due to the acidic conditions used in the experiment (pH ≥ 2).

The increase in pulp density generates a decrease in the dissolved arsenic concentration. However, this decrease is greater in runs where air was not injected to the system.

The maximum rate of arsenic dissolution in the concentrate was found using small surface area of particle exposure, low pulp density, injecting air and adding 9 K medium to the system.

The effect of addition of ferric chloride during the arsenic bioleaching resulted in a decrease of the solubilized arsenic in the system.

The presence of CO_2 _according to the results is associated to the decrease in arsenic dissolution. Further studies oriented to establish the relationship between the dissolved oxygen and the concentration of CO_2 _in the system are needed in order to elucidate this effect.

Arsenic dissolution was increased with the aeration of the system. The availability of oxygen in the medium decreases when the pulp density increases and also when CO_2 _is admitted into the system. A study using dissolved oxygen measurements is needed in the future to determine the effect of air during the arsenic bioleaching.

## Methods

### Chemical and mineralogical analysis

The flotation concentrate was obtained from La Soledad mine (Parral, Chihuahua, México). Chemical analysis was carried out by atomic absorption spectrometry through AAS (GBC Avante Σ), arsenic was determined by AAS Hydride System. The major phases in the concentrate were determined by X-ray diffraction (Siemens D5000). Mineral samples were mounted in polyester resin blocks using approximately 0.2 g per mount and surface was polished. Mounts were examined using a microscope (Olympus AX70) and photographs were taken at various sites of each sample.

### Design of experiments

One of the most useful types of multifactor experiment is the 2^*k *^factorial series. In this series, there are *k *factors each at two levels. Hence there are a total of 2^*k *^treatments in the full factorial set. This series is particularly useful in the exploratory stages of an investigation because it permits the examination of a fairly large number of factors and their interactions in a trial of reasonable size [54].

In almost all experiments the investigator would like to reduce the number of observations required for a complete factorial. If certain assumptions can be met the use of fractional factorials is a most efficient technique to reduce the number of observations and still obtain the desired information. The usual fractional factorial is still orthogonal, which means that certain effects are estimated independently of one other [55].

A major use of fractional factorial designs is in screening experiments. These are experiments in which many factors are considered with the purpose to identifying only the important variables that affect the response and their interactions. The factors that are identified as important are then investigated more thoroughly in subsequent experiments. A fractional factorial of the 2^*k *^design containing 2^*k-p *^runs is called a 1/2^*p *^fraction of the 2^*k *^or, more simply, a 2^*k-p *^fractional factorial design. It is possible to construct this type of designs for investigating up to *k *= *N-1 *factors in only *N *runs. If *k *= *N-1 *the fractional factorial design is said to be saturated. Of particular importance is a very useful saturated fractional factorial design for studying seven factors in eight runs; that is, the 2^7–4 ^design. This design is a one-sixteenth fraction of the 2^7 ^[56].

This was the design used in this work to test the factors influencing the biooxidative treatment. The objective was to maximize the arsenic solubilization. The factors, levels and runs are presented in Table 3.

### Factors and levels in experimental runs

#### Pulp density

The bioleaching experiments were carried out using two solid concentrations. The low level for pulp density was settled as 10 % w/v; the high level as 20% w/v.

#### Surface area

The lead concentrate was washed with distilled water; the mineral suspension was wet sieved using a 75 *μ*m sieve in order to obtain two fractions. Both of them were dried and analyzed for specific surface area by a laser scattering particle sizer (Malvern Master Sizer 2000). The specific surface area for fractions >75 *μ*m and <75 *μ*m were 0.42 and 1.65 m^2^/g and were established as low and high levels respectively.

#### Ferric chloride

Addition of ferric chloride at concentration of 150 mg per liter of liquid medium was settled as high level, no ferric chloride addition was low level.

#### Carbon dioxide

Low level: no carbon dioxide bubbling; high level: carbon dioxide flow from a compressed cylinder, injected into the mixture at a rate of 0.2 vol/vol min^-1^.

#### Air

No air bubbling was considered as low level; no sterile air bubbles injected into the liquid-concentrate mixture at a rate of 0.3 vol/vol min^-^1 was high level.

#### 9 K Medium

Pure distilled water as culture medium was established as low level; the use of 9 K medium [51], which contained (per liter of distilled water) 3.0 g of (NH_4_)_2_SO_4_, 0.5 g of MgSO_4_·7H_2_O, 0.1 g of KCl, 0.5 g of K_2_PO_4_, 0.01 g of Ca(NO_3_)_2 _was settled as high level. The pH was adjusted to 5.7 with sulfuric acid.

#### Strains

A native *Acidithiobacillus ferrooxidans *wild type strain termed T1 [57], isolated from a domestic mining site acid drainage, was used as a low level; and an arsenic-resistant strain, called T18, derived from T1 by serial transfers to flasks containing increasing arsenic amounts, was used as high level. T18 is able to grow at arsenic concentration as high as 1800 mg l^-1 ^[57]. The strains were cultured in a rotary shaker incubator (30°C, 175 rpm), in a medium containing 44.22 g FeSO_4_, 3.0 g (NH_4_)_2_SO_4_, 0.5 g KH_2_PO_4_, 0.5 g MgSO_4· _7H_2_O, 0.1 g KCl, 0.01 g Ca(NO_3_)_2 _per liter, adjusted to pH 2.0 with sulfuric acid. After bacterial growth for ten days, cultures were filtered and the clear liquid was used as inoculum (20% v/v).

### Conditions of cultivation and sampling

The eight biooxidation runs (Table 3) were conducted in 1000 ml Pyrex culture flasks containing 500 ml of mixture, placed in a rotary shaker incubator (30°C, 175 rpm). The pH was maintained to 2.0 with sulfuric acid. The experiment was monitored each 48 h. After a short period for sedimentation of solid particles, each flask was sampled extracting 2 ml of clear leachate in which total arsenic, lead and iron concentrations were determined. The liquid extracted was compensated by the addition of distilled water or 9 K medium. Little hoses were submerged in liquid mixture to inject compressed air, carbon dioxide, or both. The experiment was carried out for 28 days; this is twice the time needed to reach the stability of arsenic concentration in a previous laboratory test.

At the end of the experiment the resulting pulp in each run was filtered and the bioleached mineral was washed with distilled water and dried in a stove at 40°C. Two grams of this material were taken and were submitted to digestion using 10 ml of HCl 0.6 N during 3 hours at room temperature. The digested mineral was filtered, washed with distilled water and dried at 40°C. In both cases the arsenic content was determined through chemical analysis.

### Data analysis

Table 3 shows the saturated experimental design used to carry out the experiment, the eight trials provide a total of seven degrees of freedom for the entire experiment, allocated to seven columns of two levels, each column having one factor assigned (Pulp density, Surface area, Ferric chloride, Carbon dioxide, Air, 9 K medium, Strain). All columns provide four tests under the low level of the factor and four tests under the high level of the factor. This is one of the features that provides the orthogonality among all the columns (factors) [58]. Orthogonality permits the comparison between low and high levels for each factor in their ability to dissolve arsenic. Comparison was performed by fitting a multiple regression model to arsenic dissolution data, to make the model function, all seven factors were treated as dummy variables [59] taking the low level as 0 and high level as 1 (Table 7). Time in days was included in the model as the only true quantitative factor. Model fitted is:

*Arsenic *= *β_0 _*+ *β_1 _Pulp Density *+ *β_2 _Surface Area *+ *β_3 _Ferric chloride *+ *β_4 _Carbon dioxide *+ *β_5 _Air *+ *β_6 _9 K Medium *+ *β_7 _Strain *+ *β_8 _Days*.     (8)

Where

*Arsenic *Expected value of arsenic concentration in mg l^-1 ^in leachate

*β_0_*, *β_1_*,... *β_8 _*Regression coefficients

As the experiment was monitored each 48 hours, and arsenic concentration was determined in leachate, arsenic dissolution data gathered during the bioleaching experiment were analyzed as time series (time as independent variable) by multiple regression to fit the third order polynomial model:

*Arsenic *= *β*_0 _+ *β*_1_*t *+ *β*_2_*t*^2 ^+ *β*_3_*t*^3 ^    (9)

Where

*Arsenic *Expected value of arsenic concentration in mg l^-1 ^in leachate

*t *Time in days of bioleaching

*β_0_*, *β_1_*, *β_2_*, *β_3 _*Regression coefficients

### Software

Analytical procedures and graphing was performed using MS Excel or Minitab 13.0

## Authors' contributions

MM designed and performed the experimental runs and prepared the manuscript. ME performed the statistical analysis. BP provided the bacterial strains and helped in the culture production. AL performed the technical revision of the manuscript. EO got the funds and provided the tutoring during the development of this work. All authors read and approved the final manuscript.

## Figures and Tables

**Table 1:** Mineral species and associations

**Mineral species and associations**	**Relative proportion**
Galena (free)	50.41
Galena + Arsenopyrite	13.95
Pyrite (free)	5.42
Galena + Sphaelerite	4.60
Arsenopyrite (free)	3.87
Pyrrotite (free)	3.09
Sphaelerite	3.09
Galena + Pyrite	2.33
Galena + Sphaelerite + Arsenopyrite	1.55
Galena + Arsenopyrite with inclusions of Galena	1.55
Other complex associations	10.14

**Table 2:** Arsenic content in mineral residue before and after digestion with hydrochloric acid

**Run**	**Residue before digestion % arsenic**	**Residue after digestion % arsenic**	**Difference % arsenic**
1	3.5208	2.6332	0.8876
2	3.5751	2.7105	0.8646
3	3.6283	3.0034	0.6249
4	3.6452	2.9911	0.6541
5	3.5291	2.5907	0.9384
6	3.5325	2.5821	0.9504
7	3.6334	2.9436	0.6898
8	3.6021	2.9784	0.6237

**Table 3:** The 2^7–4 ^design used.

***Pattern***	***Run***	***Pulp Density %***	***Surface Area***	***Ferric Chloride***	***Carbon Dioxide***	***Air***	***9 K Medium***	***Strain***
----+++	1	10	Low	No added	No added	Added	Added	T18
--++--+	2	10	Low	Added	Added	No added	No added	T18
-+-+-+-	3	10	High	No added	Added	No added	Added	T1
-++-+--	4	10	High	Added	No added	Added	No added	T1
+--++--	5	20	Low	No added	Added	Added	No added	T1
+-+--+-	6	20	Low	Added	No added	No added	Added	T1
++----+	7	20	High	No added	No added	No added	No added	T18
+++++++	8	20	High	Added	Added	Added	Added	T18

**Table 4:** Regression analysis results

**Predictor**	**Coefficient**	**T value**	**Probability**
Constant	52.0270	6.76	0.000
Pulp density %	-66.3470	-14.05	0.000
Surface area	-32.5570	-6.89	0.000
Ferric chloride	-21.0730	-4.46	0.000
Carbon dioxide	-12.5350	-2.65	0.009
Air	29.3000	6.20	0.000
9 K medium	29.3010	6.20	0.000
Strain	3.0020	0.64	0.526
Days	3.9396	14.41	0.000

**Table 5:** Analysis of variance for interaction Pulp density – Air

**Source**	**DF**	**SS**	**MS**	**F**	**P**
Air	1	4714	4714	1.92	0.168
Pulp Density	1	132058	132058	53.84	0.000
Interaction	1	25775	25775	10.50	0.002
Error	116	284501	2453		
Total	119	447028			

DF: Degrees of Freedom; SS: Sum of Squares; MS: Mean Square; F: Fisher Statistic; P: Probability

**Table 6:** Analysis of variance for interaction Carbon dioxide – Air

**Source**	**DF**	**SS**	**MS**	**F**	**P**
Air	1	4714	4714	1.92	0.168
Carbon dioxide	1	25755	25775	10.50	0.002
Interaction	1	132058	132058	53.84	0.000
Error	116	284501	2453		
Total	119	447028			

DF: Degrees of Freedom; SS: Sum of Squares; MS: Mean Square; F: Fisher Statistic; P: Probability

**Table 7:** Factors coded as dummy variables

***Pattern***	***Run***	***Pulp Density***	***Surface Area***	***Ferric Chloride***	***Carbon Dioxide***	***Air***	***9 K medium***	***Strain***	***Results As mg/l***
----+++	1	0	0	0	0	1	1	1	R1
--++--+	2	0	0	1	1	0	0	1	R2
-+-+-+-	3	0	1	0	1	0	1	0	R3
-++-+--	4	0	1	1	0	1	0	0	R4
+--++--	5	1	0	0	1	1	0	0	R5
+-+--+-	6	1	0	1	0	0	1	0	R6
++----+	7	1	1	0	0	0	0	1	R7
+++++++	8	1	1	1	1	1	1	1	R8

Results of As in leachate (mg/l) were determined each 48 hours, to finally have the results of this table 15 times during the 28 days of the experiment
